# Supplementary material for: Interpregnancy intervals and adverse birth outcomes in high-income countries: An international cohort study
Source: PLoS One. 2021 Jul 19;16(7):e0255000. doi: 10.1371/journal.pone.0255000 (PMC8289039; doi:10.1371/journal.pone.0255000)
Supplement: S1 Fig — (DOCX) [file pone.0255000.s002.docx]

**S1 Fig**. Flowchart of study inclusions and exclusions –Australia, Finland, Norway and California

**Eligible**: singleton births

(Australia= 2,984,955, Finland=1,797,768, Norway=2,024,816, California =4,364,708)

***Multiple births********
(Australia= 89,270, Finland =52,678, Norway=63,437)

**Eligible:**

(Australia=2,974,162, Finland =1,788,614, Norway=1,920,081, California =4,136,278)

**B*irths with birthweight null***

(Australia= 925, Finland =483, Norway= 1,678, California =15,587)

**Eligible:**

(Australia= 2,971,534, Finland =1,788,027,
Norway= 1,918,265, California =4,120,657)

***Births with gestational age null, <22 weeks or >44 weeks***

(Australia= 10,793, Finland =9,154, Norway=104, 735, California=228,430)

**Eligible**:

(Australia=2,973,406, Finland= 1788,571, Norway=1,920,081, California =4,136,278)

**Eligible**:

(Australia=2,972,481, Finland =1,788,088,
Norway = 1,918,403, California =4,120,691)

***Births with maternal age null***

(Australia= 756, Finland =43, Norway=0, California =0)

***Births with undetermined /unknown sex***

(Australia= 947, Finland =61, Norway=138, California=34)

**Included (between-women analysis):**

**5,521,206 births (3,849,193 women)**

(Australia births =1,331,560, women=899,102)

(Finland births=866,389, women=559,608)

(Norway =923,333, women=649,982)

(California =2,399,924, women=1,740,499)

***Births with no IPI***

(Australia= 1,639,974, Finland =349,513, Norway=994,932, California =1,720,733)

**Included (within-women analysis):**

**2,905,703 births (1,233,688 women)**

(Australia births =739,097, women= 306,639)

(Finland births=509,033, women=202,252)

(Norway births =493,121, women= 219,770)

(California births= 1,164,452, women=505,027)

***Births and women with <2 IPIs***

(Australia births=592,463, women=592,463)

(Finland births=357,356, women=357,356)

(Norway = 430,212, women=430,212)

(California = 1,235,472, women=1,234,472)

**Total number of births in the cohorts (11,383,557)**(Australia =3,074,232, Finland=1,850,446,
Norway =2,094,171, California= 4,364,708)*

***Missing women’s ID*******
(Norway= 5,918)

*California began with births to women who had their first birth in California. Women’s ID missing only in Norway. **For California, the cohort accessed singleton births at the beginning.

**Excluded sample**
