# Supplementary material for: Interpregnancy intervals and adverse birth outcomes in high-income countries: An international cohort study
Source: PLoS One. 2021 Jul 19;16(7):e0255000. doi: 10.1371/journal.pone.0255000 (PMC8289039; doi:10.1371/journal.pone.0255000)
Supplement: S2 Fig — (DOCX) [file pone.0255000.s003.docx]

# **S2 Fig.** Adjusted odds ratios for the between-women analysis for the association between interpregnancy interval and preterm birth as compared to 18-23 months of interpregnancy interval by country

.

.

.

.

.

.

**<6 months**

Australia

Finland

Norway

California

**Subtotal (I^2^ = 98.6%, p = 0.00)**

**6-11 months**

Australia

Finland

Norway

California

**Subtotal (I^2^ = 85.1%, p = 0.00)**

**12-17 months**

Australia

Finland

Norway

California

**Subtotal (I^2^ = 88.6%, p = 0.00)**

**24-59 months**

Australia

Finland

Norway

California

**Subtotal (I^2^ = 52.5%, p = 0.10)**

**60-119 months**

Australia

Finland

Norway

California

**Subtotal (I^2^ = 94.2%, p = 0.000)**

**≥120 months**

Australia

Finland

Norway

California

**Subtotal (I^2^ = 89.9%, p = 0.00)**

**IPI by country**

1.68 (1.63, 1.73)

1.62 (1.53, 1.70)

2.06 (1.96, 2.17)

1.42 (1.39, 1.45)

1.68 (1.45, 1.95)

1.13 (1.10, 1.16)

1.05 (1.01, 1.10)

1.17 (1.12, 1.22)

1.16 (1.14, 1.18)

1.13 (1.09, 1.17)

0.99 (0.96, 1.02)

0.99 (0.95, 1.03)

1.02 (0.98, 1.06)

1.06 (1.05, 1.08)

1.02 (0.97, 1.06)

1.16 (1.13, 1.19)

1.12 (1.08, 1.16)

1.11 (1.08, 1.15)

1.12 (1.10, 1.14)

1.13 (1.11, 1.15)

1.59 (1.55, 1.64)

1.41 (1.35, 1.47)

1.51 (1.45, 1.57)

1.41 (1.39, 1.44)

1.48 (1.39, 1.57)

2.14 (2.03, 2.27)

1.73 (1.61, 1.86)

1.91 (1.81, 2.02)

1.82 (1.76, 1.88)

1.90 (1.75, 2.06)

**aOR (95% CI)***

25.20

24.70

24.74

25.36

100.00

26.42

22.25

22.62

28.71

100.00

25.88

22.80

23.09

28.23

100.00

27.64

17.86

20.46

34.04

100.00

25.23

23.89

24.58

26.30

100.00

24.92

23.17

24.92

26.99

100.00

**Weight (%)****

1.68 (1.63, 1.73)

1.62 (1.53, 1.70)

2.06 (1.96, 2.17)

1.42 (1.39, 1.45)

1.68 (1.45, 1.95)

1.13 (1.10, 1.16)

1.06 (1.01, 1.10)

1.17 (1.12, 1.22)

1.16 (1.14, 1.18)

1.13 (1.09, 1.17)

0.99 (0.96, 1.02)

0.99 (0.95, 1.03)

1.02 (0.98, 1.06)

1.06 (1.05, 1.08)

1.02 (0.97, 1.06)

1.16 (1.13, 1.19)

1.12 (1.08, 1.16)

1.11 (1.08, 1.15)

1.12 (1.10, 1.14)

1.13 (1.11, 1.15)

1.59 (1.55, 1.64)

1.41 (1.35, 1.47)

1.51 (1.45, 1.57)

1.41 (1.39, 1.44)

1.48 (1.39, 1.57)

2.14 (2.03, 2.27)

1.73 (1.61, 1.86)

1.91 (1.81, 2.02)

1.82 (1.76, 1.88)

1.90 (1.75, 2.06)

25.20

24.70

24.74

25.36

100.00

26.42

22.25

22.62

28.71

100.00

25.88

22.80

23.09

28.23

100.00

27.64

17.86

20.46

34.04

100.00

25.23

23.89

24.58

26.30

100.00

24.92

23.17

24.92

26.99

100.00

1

.5

1

1.5

2

IPI - interpregnancy interval. *adjusted odds ratios (aOR) and corresponding 95% confidence intervals, adjusted for maternal age, parity, and birth year; the reference IPI category is 18-23 months. **Weights are derived from inverse-variance.
