# Supplementary material for: Interpregnancy intervals and adverse birth outcomes in high-income countries: An international cohort study
Source: PLoS One. 2021 Jul 19;16(7):e0255000. doi: 10.1371/journal.pone.0255000 (PMC8289039; doi:10.1371/journal.pone.0255000)
Supplement: S3 Fig — (DOCX) [file pone.0255000.s004.docx]

# **S3 Fig**. Adjusted odds ratios for the between-women analysis for the association between interpregnancy interval and spontaneous preterm birth as compared to 18-23 months of interpregnancy interval by country.

.

.

.

.

.

.

**<6 months**

Australia

Finland

Norway

California

**Subtotal (I^2^ = 94.0%, p = 0.00)**

**6-11 months**

Australia

Finland

Norway

California

**Subtotal (I^2^ = 73.5%, p = 0.01)**

**12-17 months**

Australia

Finland

Norway

California

**Subtotal (I^2^ = 0.0%, p = 0.98)**

**24-59 months**

Australia

Finland

Norway

California

**Subtotal (I^2^ = 74.5%, p = 0.010)**

**60-119 months**

Australia

Finland

Norway

California

**Subtotal (I^2^ = 79.3%, p = 0.00)**

**≥120 months**

Australia

Finland

Norway

California

**Subtotal (I^2^ = 68.5%, p = 0.02)**

**IPI by country**

1.93 (1.85, 2.01)

1.77 (1.66, 1.88)

2.04 (1.92, 2.18)

1.67 (1.62, 1.72)

1.84 (1.68, 2.02)

1.22 (1.18, 1.27)

1.14 (1.09, 1.20)

1.22 (1.16, 1.28)

1.15 (1.12, 1.18)

1.18 (1.14, 1.23)

1.03 (0.99, 1.07)

1.04 (0.99, 1.09)

1.04 (0.99, 1.09)

1.03 (1.00, 1.06)

1.03 (1.01, 1.05)

1.13 (1.10, 1.17)

1.15 (1.10, 1.20)

1.06 (1.02, 1.11)

1.15 (1.12, 1.17)

1.12 (1.09, 1.16)

1.56 (1.50, 1.63)

1.45 (1.37, 1.52)

1.40 (1.34, 1.47)

1.52 (1.48, 1.56)

1.48 (1.42, 1.55)

1.99 (1.84, 2.16)

1.76 (1.62, 1.92)

1.78 (1.64, 1.90)

1.96 (1.88, 2.05)

1.88 (1.77, 2.00)

**aOR (95% CI)***

25.69

24.08

23.97

26.25

100.00

26.58

22.02

21.63

29.76

100.00

28.06

15.22

15.22

41.50

100.00

26.21

21.66

22.12

30.01

100.00

25.09

22.26

23.77

28.88

100.00

22.74

21.64

24.18

31.43

100.00

**Weight (%)****

1.93 (1.85, 2.01)

1.77 (1.66, 1.88)

2.04 (1.92, 2.18)

1.67 (1.62, 1.72)

1.84 (1.68, 2.02)

1.22 (1.18, 1.27)

1.14 (1.09, 1.20)

1.22 (1.16, 1.28)

1.15 (1.12, 1.18)

1.18 (1.14, 1.23)

1.03 (0.99, 1.07)

1.04 (0.99, 1.09)

1.04 (0.99, 1.09)

1.03 (1.00, 1.06)

1.03 (1.01, 1.05)

1.13 (1.10, 1.17)

1.15 (1.10, 1.20)

1.06 (1.02, 1.11)

1.15 (1.12, 1.17)

1.12 (1.09, 1.16)

1.56 (1.50, 1.63)

1.45 (1.37, 1.52)

1.40 (1.34, 1.47)

1.52 (1.48, 1.56)

1.48 (1.42, 1.55)

1.99 (1.84, 2.16)

1.76 (1.62, 1.92)

1.78 (1.64, 1.90)

1.96 (1.88, 2.05)

1.88 (1.77, 2.00)

25.69

24.08

23.97

26.25

100.00

26.58

22.02

21.63

29.76

100.00

28.06

15.22

15.22

41.50

100.00

26.21

21.66

22.12

30.01

100.00

25.09

22.26

23.77

28.88

100.00

22.74

21.64

24.18

31.43

100.00

1

.5

1

1.5

2

IPI - interpregnancy interval. *Weights are derived from inverse-variance. **adjusted odds ratios (aOR) and corresponding 95% confidence intervals, adjusted for maternal age, parity, and birth year; the reference IPI category is 18-23 months.
