# Supplementary material for: Interpregnancy intervals and adverse birth outcomes in high-income countries: An international cohort study
Source: PLoS One. 2021 Jul 19;16(7):e0255000. doi: 10.1371/journal.pone.0255000 (PMC8289039; doi:10.1371/journal.pone.0255000)
Supplement: S4 Fig — (DOCX) [file pone.0255000.s005.docx]

# **S4 Fig.** Adjusted odds ratios for the between-women analysis for the association between interpregnancy interval and small-for-gestational age birth as compared to 18-23 months of interpregnancy interval by country.

.

.

.

.

.

.

<**6 months**

Australia

Finland

Norway

California

**Subtotal (I^2^ = 98.7%, p = 0.00)**

**6-11 months**

Australia

Finland

Norway

California

**Subtotal (I^2^ = 95.7%, p = 0.00)**

**12-17 months**

Australia

Finland

Norway

California

**Subtotal (I^2^ = 31.8%, p = 0.22)**

**24-59 months**

Australia

Finland

Norway

California

**Subtotal (I^2^ = 87.6%, p = 0.00)**

**60-119 months**

Australia

Finland

Norway

California

**Subtotal (I^2^ = 98.2%, p = 0.00)**

**≥120 months**

Australia

Finland

Norway

California

**Subtotal (I^2^ = 95.1%, p = 0.00)**

**IPI by country**

1.10 (1.07, 1.13)

1.07 (1.03, 1.12)

1.28 (1.22, 1.35)

1.42 (1.39, 1.46)

1.21 (1.04, 1.41)

0.99 (0.96, 1.01)

0.95 (0.92, 0.98)

1.08 (1.05, 1.12)

1.08 (1.06, 1.10)

1.02 (0.96, 1.09)

0.98 (0.96, 1.00)

0.98 (0.95, 1.01)

1.02 (0.99, 1.05)

1.00 (0.98, 1.02)

0.99 (0.98, 1.01)

1.18 (1.15, 1.20)

1.09 (1.06, 1.12)

1.11 (1.08, 1.14)

1.13 (1.11, 1.15)

1.13 (1.09, 1.16)

1.65 (1.61, 1.69)

1.35 (1.31, 1.40)

1.42 (1.37, 1.46)

1.37 (1.34, 1.40)

1.44 (1.32, 1.57)

2.08 (1.98, 2.19)

1.72 (1.63, 1.81)

1.83 (1.75, 1.92)

1.64 (1.59, 1.70)

1.81 (1.63, 2.01)

**aOR (95% CI)**

25.14

24.91

24.73

25.23

100.00

25.24

24.53

24.60

25.62

100.00

28.81

19.23

18.05

33.91

100.00

25.75

23.70

24.11

26.43

100.00

25.01

24.68

24.81

25.49

100.00

24.74

24.68

24.92

25.65

100.00

**Weight (%)****

1.10 (1.07, 1.13)

1.07 (1.03, 1.12)

1.28 (1.22, 1.35)

1.42 (1.39, 1.46)

1.21 (1.04, 1.41)

0.99 (0.96, 1.01)

0.95 (0.92, 0.98)

1.08 (1.05, 1.12)

1.08 (1.06, 1.10)

1.02 (0.96, 1.09)

0.98 (0.96, 1.00)

0.98 (0.95, 1.01)

1.02 (0.99, 1.05)

1.00 (0.98, 1.02)

0.99 (0.98, 1.01)

1.18 (1.15, 1.20)

1.09 (1.06, 1.12)

1.11 (1.08, 1.14)

1.13 (1.11, 1.15)

1.13 (1.09, 1.16)

1.65 (1.61, 1.69)

1.44 (1.32, 1.57)

2.08 (1.98, 2.19)

1.72 (1.63, 1.81)

1.83 (1.75, 1.92)

1.64 (1.59, 1.70)

1.81 (1.63, 2.01)

25.14

24.91

24.73

25.23

100.00

25.24

24.53

24.60

25.62

100.00

28.81

19.23

18.05

33.91

100.00

25.75

23.70

24.11

26.43

100.00

25.01

24.68

24.81

25.49

100.00

24.74

24.68

24.92

25.65

100.00

1

.5

1

1.5

2

#

IPI - interpregnancy interval. *adjusted odds ratios (aOR) and corresponding 95% confidence intervals, adjusted for maternal age, parity, and birth year; the reference IPI category is 18-23 months. **Weights are derived from inverse-variance.
