# Supplementary material for: Interpregnancy intervals and adverse birth outcomes in high-income countries: An international cohort study
Source: PLoS One. 2021 Jul 19;16(7):e0255000. doi: 10.1371/journal.pone.0255000 (PMC8289039; doi:10.1371/journal.pone.0255000)
Supplement: S1 Table — (DOCX) [file pone.0255000.s006.docx]

# **S1 Table.** Maternal characteristics at study entry (first birth in the included cohort) * for the between-women analyses across the four countries (n=3,849,191).

| **Characteristics** | **Australia** | **Finland** | **Norway** | **California** |
| --- | --- | --- | --- | --- |
|  | **N (%)** | **N (%)** | **N (%)** | **N (%)** |
| **Total women** | 899,102 | 559,608 | 649,982 | 1,740,499 |
| **Parity**** | | | | |
| 1 | 780,258 (86.8) | 503,876 (90.0) | 584,196 (89.9) | 1,704,824 (98.0) |
| 2 | 74,916 (8.3) | 35,290 (6.3) | 48,230 (7.4) | 34,078 (1.9) |
| ≥3 | 43,928 (4.9) | 20,442 (3.7) | 17,556 (2.7) | 1,597 (0.1) |
| **Maternal age (years)** | | |  |  |
| <20 | 17,488 (2.0) | 3,580 (0.6) | 3,040 (0.5) | 86,762 (5.0) |
| 20-24 | 129,643 (14.4) | 77,090 (13.8) | 91,851 (14.1) | 404,395 (23.2) |
| 25-29 | 259,226 (28.8) | 192,085 (34.3) | 242,649 (37.3) | 468,096 (26.9) |
| 30-34 | 308,221 (34.3) | 192,833 (34.5) | 221,372 (34.1) | 477,219 (27.4) |
| 35-39 | 155,446 (17.3) | 79,204 (14.2) | 79021 (12.2) | 253,716 (14.6) |
| ≥40 | 29,078 (3.2) | 14,816 (2.7) | 12,049 (1.9) | 50,311 (2.9) |
| **Birth year** | | | | |
| 1980-1989 | 64,823 (7.2) | 17,139 (3.1) | 128,721 (19.8) | - |
| 1990-1999 | 190,241 (21.2) | 212,512 (38.0) | 199,887 (30.8) | 512,327 (29.4) |
| 2000-2009 | 373,371 (41.5) | 182,857 (32.7) | 198,469 (30.5) | 942,930 (44.2) |
| 2010 or later | 270,667 (30.1) | 147,100 (26.2) | 122,905 (18.9) | 285,242 (16.4) |
| **Socioeconomic index for areas (quintile)** | | | | |
| Lowest | 168,178 (18.7) | N/A | N/A | N/A |
| Low | 165,733 (18.4) | N/A | N/A | N/A |
| Middle | 168,370 (18.7) | N/A | N/A | N/A |
| High | 173,268 (19.3) | N/A | N/A | N/A |
| Highest | 178,071 (19.8) | N/A | N/A | N/A |
| Other/unknown/missing | 45,482 (5.06) | N/A | N/A | N/A |
| **Maternal occupation** | | | | |
| Upper-white collar worker | N/A | 94,765 | N/A | N/A |
| Lower-white collar worker | N/A | 215,109 | N/A | N/A |
| Blue collar worker | N/A | 84,304 | N/A | N/A |
| Other or unknown | N/A | 165,430 | N/A | N/A |
| **Highest level of maternal education** | | | | |
| Some high school or less | N/A | N/A | N/A | 225,032 (12.9) |
| High school diploma/Equivalent | N/A | N/A | N/A | 490,455 (28.2) |
| Some college | N/A | N/A | N/A | 451,004 (25.9) |
| College graduate or more | N/A | N/A | N/A | 543,605 (31.2) |
| Missing | N/A | N/A | N/A | 30,403 (1.8) |
| **Number of IPIs** | | | | |
| 1 | 59,2463 (65.9) | 357,739 (63.9) | 430,212 (66.1) | 1,235,472 (71.0) |
| 2 | 220,175 (24.5) | 142,471 (25.5) | 177,636 (27.3) | 385,449 (22.2) |
| ≥3 | 86,464 (9.7) | 59,398 (10.6) | 42,143 (6.6) | 119,57 (6.8) |

*Study entry is defined as the earliest birth (second birth) at which the women appeared within the included cohort during the study period **Women parity at birth when entering to the cohort (i.e the second birth after interval). IPI-Interpregnancy intervals. N/A- data were not available in these countries/sites.
