# Supplementary material for: Interpregnancy intervals and adverse birth outcomes in high-income countries: An international cohort study
Source: PLoS One. 2021 Jul 19;16(7):e0255000. doi: 10.1371/journal.pone.0255000 (PMC8289039; doi:10.1371/journal.pone.0255000)
Supplement: S2 Table — (DOCX) [file pone.0255000.s007.docx]

# **S2 Table.** Distributions of interpregnancy intervals by adverse birth outcomes in the cohort for the within-women analyses across the four countries.

| **Outcome by country** | **Interpregnancy interval** | | | | | | |  |
| --- | --- | --- | --- | --- | --- | --- | --- | --- |
|  | **<6 months** | **6-11 months** | **12-17 months** | **18-23 months** | **24-59 months** | **60-119 months** | ≥**120 months** | **Total (%)** |
| **PTB** | | | | | | | | |
| **Australia** | 5,294 (9.65) | 8,234 (6.19) | 7,544 (5.24) | 5,717 (5.30) | 13,649 (5.91) | 4,642 (7.73) | 819 (9.82) | 45,899 (6.21) |
| **Finland** | 1,690 (5.54) | 3,480 (3.69) | 3,220 (3.42) | 2,403 (3.59) | 5,963 (3.73) | 2,630 (4.81) | 553 (6.32) | 19,939 (3.92) |
| **Norway** | 1,618 (8.69) | 2,911 (4.93) | 3,140 (4.18) | 2,725 (4.07) | 8,633 (4.44) | 4,018 (5.91) | 830 (7.44) | 23,875 (4.84) |
| **California** | 10,401 (11.37) | 15,493 (8.34) | 15,092 (8.12) | 11,350 (7.56) | 33,496 (8.08) | 12,484 (9.31) | 1,801 (11.22) | 100,117 (8.60) |
| **Spontaneous PTB** | | | | | | | | |
| **Australia** | 3,523 (6.740) | 5,061 (3.94) | 4,320(3.10) | 3,103 (2.98) | 6,937 (3.13) | 2,231 (3.91) | 354 (4.57) | 25,529 (3.59) |
| **Finland** | 1,345 (4.65) | 2,722 (3.03) | 2,389 (2.69) | 1,658 (2.64) | 4,225 (2.86) | 1,833 (3.68) | 374 (4.75) | 14,546 (3.06) |
| **Norway** | 1,021 (5.80) | 1,867 (3.26) | 2,001 (2.58) | 1,684 (2.58) | 4,877 (2.58) | 2,136 (3.26) | 416 (3.91) | 14,002 (2.93) |
| **California** | 4,909 (5.71) | 6,417 (3.93) | 6,141 (3.47) | 4,727 (3.29) | 14,796 (3.74) | 6,226 (3.87) | 990 (6.49) | 44,206 (3.99) |
| **SGA** | | | | | | | | |
| **Australia** | 4,995 (9.11) | 9,999 (7.52) | 10,334 (7.18) | 7,693(7.13) | 18,747(8.12) | 6,124 (10.19) | 962 (11.54) | 58,854 (7.96) |
| **Finland** | 2,173 (7.13) | 5,920 (6.72) | 5989 (6.37) | 4240 (6.33) | 10,870 (6.81) | 4,466 (8.18) | 870 (11.09) | 34,628 (6.80) |
| **Norway** | 1,512 (8.12) | 3,993 (6.77) | 4,678 (6.22) | 4,114 (6.15) | 12,454 (6.41) | 5,234 (7.70) | 1,113 (9.98) | 33,098 (6.71) |
| **California** | 9,358 (10.23) | 12,880 (7.47) | 12,611 (6.79) | 10,067 (6.67) | 29,732 (7.17) | 10,510 (7.84) | 1,460 (9.09) | 86,568 (7.43) |

*Numbers in the brackets are column percentages calculated from the samples in each country.
