# Supplementary material for: Interpregnancy intervals and adverse birth outcomes in high-income countries: An international cohort study
Source: PLoS One. 2021 Jul 19;16(7):e0255000. doi: 10.1371/journal.pone.0255000 (PMC8289039; doi:10.1371/journal.pone.0255000)
Supplement: S3 Table — (DOCX) [file pone.0255000.s008.docx]

# **S3 Table**. Sensitivity analysis - Association between interpregnancy interval and adverse birth outcomes in the between-women* and within-women analyses** adjusted for parity, maternal age, time period, and socioeconomic status*** across the four countries.

| **Outcome by country** | **Interpregnancy interval** | | | | | | |
| --- | --- | --- | --- | --- | --- | --- | --- |
|  | **<6 months** | **6-11 months** | **12-17 months** | **18-23 months** | **24-59 months** | **60-119 months** | **>120months** |
| **PTB aOR (95% CI)** | | | | | | | |
| **Australia** | | | | | | | |
| between-women | 1.67 (1.61, 1.72) | 1.13 (1.10, 1.16) | 0.99 (0.96,1.02) | Ref | 1.15 (1.13, 1.18) | 1.55 (1.50, 1.60) | 2.07 (1.95, 2.19) |
| within-women | 1.22 (1.15, 1.29) | 1.09 (1.03, 1.15) | 0.99 (0.94, 1.05) | Ref | 1.06 (1.01, 1.11) | 1.33 (1.26, 1.42) | 1.68 (1.49, 1.90) |
| **Finland** | | | | | | | |
| between-women | 1.60 (1.52, 1.69) | 1.05 (1.01, 1.10) | 0.99 (0.95, 1.03) | Ref | 1.12 (1.08, 1.16) | 1.41 (1.35, 1.47) | 1.72 (1.60, 1.85) |
| within-women | 1.00 (0.91, 1.09) | 1.02 (0.95, 1.10) | 0.95 (0.88, 1.02) | Ref | 1.00 (0.94, 1.07) | 1.26 (1.16, 1.37) | 1.67 (1.43, 1.94) |
| **California** | | | | | | | |
| Unmatched | 1.40 (1.37,1.43) | 1.15 (1.13,1.18) | 1.07 (1.05,1.09) | Ref | 1.09 (1.08,1.11) | 1.32 (1.30,1.35) | 1.63 (1.58,1.69) |
| Matched | 1.13 (1.09, 1.18) | 1.10 (1.06, 1.14) | 1.07 (1.03, 1.11) | Ref | 1.00 (0.97,1.03) | 1.15 (1.11,1.19) | 1.36 (1.26,1.48) |
| **Spontaneous PTB** | | | | | | | |
| **Australia** | | | | | | | |
| between-women | 1.91 (1.83, 1.99) | 1.22 (1.18, 1.27) | 1.02 (0.99, 1.06) | Ref | 1.12 (1.09, 1.16) | 1.52 (1.46, 1.59) | 1.93 (1.83, 1.99) |
| within-women | 1.65 (1.53, 1.78) | 1.31 (1.22, 1.40) | 1.05 (0.98, 1.12) | Ref | 0.95 (0.89, 1.01) | 1.09 (1.00, 1.18) | 1.29 (1.09, 1.53) |
| **Finland** | | | | | | | |
| between-women | 1.76 (1.66, 1.87) | 1.14 (1.08, 1.20) | 1.04 (0.99, 1.09) | Ref | 1.15 (1.10, 1.21) | 1.45 (1.38, 1.53) | 1.76 (1.61, 1.91) |
| within-women | 1.32 (1.18, 1.46) | 1.22 (1.12, 1.33) | 1.04 (0.96, 1.14) | Ref | 1.05 (0.97, 1.14) | 1.35 (1.22, 1.49) | 1.77 (1.47, 2.12) |
| **California** | | | | | | | |
| between-women | 1.65 (1.60,1.70) | 1.15 (1.12,1.18) | 1.03 (1.00, 1.06) | Ref | 1.13 (1.11, 1.16) | 1.45 (1.41, 1.49) | 1.81 (1.73, 1.89) |
| within-women | 1.34 (1.26,1.43) | 1.12 (1.06, 1.18) | 1.06 (1.00, 1.13) | Ref | 1.05 (1.00, 1.10) | 1.32 (1.25, 1.40) | 1.59 (1.41, 1.79) |
| **SGA** | | | | | | | |
| **Australia** |  |  |  |  |  |  |  |
| between-women | 1.09 (1.06, 1.12) | 0.99 (0.97, 1.12) | 0.98 (0.96, 1.01) | Ref | 1.16 (1.14, 1.18) | 1.56 (1.52, 1.61) | 1.93 (1.83, 2.03) |
| within-women | 0.94 (0.88, 0.99) | 0.99 (0.95, 1.04) | 1.03 (0.99, 1.08) | Ref | 1.04 (1.00, 1.08) | 1.24 (1.17, 1.31) | 1.58 (1.40, 1.78) |
| **Finland** | | | | | | | |
| between-women | 1.08 (1.01, 1.18) | 0.89 (0.84, 0.94) | 0.90 (0.85, 0.95) | Ref | 1.14 (1.08, 1.19) | 1.54 (1.45, 1.63) | 2.19 (2.00, 2.39) |
| within-women | 0.89 (0.83, 0.96) | 0.99 (0.93, 1.05) | 1.01 (0.95, 1.07) | Ref | 1.08 (1.02, 1.13) | 1.23 (1.16, 1.32) | 1.78 (1.58, 2.01) |
| **California** | | | | | | | |
| between-women | 1.41 (1.37, 1.44) | 1.08 (1.06, 1.10) | 1.01 (0.99, 1.02) | Ref | 1.11 (1.09, 1.13) | 1.30 (1.27, 1.32) | 1.51 (1.46, 1.57) |
| within-women | 1.26 (1.21,1.32) | 1.05 (1.01,1.09) | 1.00 (0.96,1.04) | Ref | 1.03 (1.00, 1.07) | 1.16 (1.11, 1.21) | 1.48 (1.36, 1.62) |

aOR- adjusted odds ratio. CI - confidence interval. IPI - interpregnancy interval. PTB - preterm birth. SGA - small for gestational age. *Odds ratios calculated using between-women analyses for women with ≥2 births/ ≥1 IPI after prognostic score adjustment for maternal age, parity, year of birth, and socioeconomic status. **Odds ratios calculated using within-women analyses for women with ≥3 births/ ≥2 IPIs after prognostic score adjustment for maternal age, parity, year of birth, and socioeconomic status.

***Socioeconomic status was collected by Australia, Finland, and California. In Australia, this was defined based on scores from the socio-economic indexes for geographical areas composite of education, skilled occupation status, and household income ; in Finland, this was defined as a proxy measure using maternal occupational status at the time of birth; in California, this was defined as a proxy measure using maternal educational attainment at the time of birth.
