# Supplementary material for: Interpregnancy intervals and adverse birth outcomes in high-income countries: An international cohort study
Source: PLoS One. 2021 Jul 19;16(7):e0255000. doi: 10.1371/journal.pone.0255000 (PMC8289039; doi:10.1371/journal.pone.0255000)
Supplement: S4 Table — (DOCX) [file pone.0255000.s009.docx]

# **S4 Table.** Sensitivity analysis - Association between interpregnancy interval and adverse birth outcomes for within-women analyses* after excluding stillbirths in the IPI calculation adjusted for parity, maternal age, time period across the four countries.

| **Outcome by country** | **Interpregnancy interval** | | | | | | |
| --- | --- | --- | --- | --- | --- | --- | --- |
|  | **<6 months** | **6-11 months** | **12-17 months** | **18-23 months** | **24-59 months** | **60-119 months** | ≥**120months** |
| **PTB** **aOR (95% CI)** | | | | | | | |
| **Australia** | 1.39 (1.31, 1.48) | 1.13 (1.08, 1.20) | 1.02 (0.97, 1.08) | Ref | 1.05 (1.01, 1.11) | 1.36 (1.28, 1.45) | 1.76 (1.56, 1.99) |
| **Finland** | 1.16 (1.05, 1.28) | 1.07 (0.99, 1.16) | 0.96 (0.89, 1.04) | Ref | 0.99 (0.92, 1.06) | 1.26 (1.15, 1.37) | 1.65 (1.40, 1.93) |
| **Norway** | 1.32 (1.19, 1.45) | 1.10 (1.01, 1.19) | 1.08 (0.99, 1.16) | Ref | 1.07 (1.00, 1.15) | 1.36 (1.26, 1.47) | 1.70 (1.50, 1.94) |
| **California** | 1.17 (1.12, 1.22) | 1.11 (1.07, 1.15) | 1.08 (1.04, 1.12) | Ref | 1.00 (0.97, 1.03) | 1.16 (1.11, 1.21) | 1.41 (1.30, 1.53) |
| **Spontaneous PTB** | | | | | | | |
| **Australia** | 1.74 (1.62, 1.88) | 1.32 (1.24, 1.41) | 1.08 (1.00, 1.15) | Ref | 0.96 (0.90, 1.02) | 1.09 (1.01, 1.19) | 1.27 (1.08, 1.51) |
| **Finland** | 1.44 (1.29, 1.61) | 1.25 (1.15, 1.37) | 1.04 (0.95, 1.14) | Ref | 1.03 (0.95, 1.12) | 1.34 (1.21, 1.48) | 1.54 (1.44, 2.11) |
| **Norway** | 1.38 (1.22, 1.56) | 1.15 (1.04, 1.27) | 1.08 (0.98, 1.18) | Ref | 0.94 (0.87, 1.02) | 1.12 (1.02, 1.24) | 1.30 (1.10, 1.53) |
| **California** | 1.36 (1.27, 1.45) | 1.13 (1.06, 1.20) | 1.06 91.00, 1.12) | Ref | 1.05 (0.99, 1.11) | 1.34 (1.26, 1.43) | 1.64 (1.46, 1.84) |
| **SGA** | | | | | | | |
| **Australia** | 0.98 (0.93, 1.04) | 1.01 (0.96, 1.05) | 1.04 (0.99, 1.09) | Ref | 1.04 (1.00, 1.08) | 1.23 (1.16, 1.30) | 1.56 (1.39, 1.76) |
| **Finland** | 0.73 (0.64, 0.84) | 0.93 (0.84, 1.04) | 0.90 (0.91, 1.00) | Ref | 1.09 (0.99, 1.20) | 1.38 (1.23, 1.54) | 1.97 (1.61, 2.40) |
| **Norway** | 0.98 (0.89, 1.07) | 1.01 (0.95, 1.09) | 0.99 (0.92, 1.05) | Ref | 1.04 (0.99, 1.10) | 1.23 (1.15, 1.32) | 1.59 (1.42, 1.78) |
| **California** | 1.28 (1.23, 1.34) | 1.06 (1.02, 1.10) | 1.00 (0.96, 1.04) | Ref | 1.03 (1.00, 1.06) | 1.17 (1.12, 1.22) | 1.51 (1.38, 1.65) |

aOR- adjusted odds ratio. CI - confidence interval. PTB - preterm birth. SGA - small for gestational age. *Odds ratios calculated using within-women analyses for women with ≥3 births/ ≥2 IPIs after prognostic score adjustment for maternal age, parity, and year of birth.
