# Supplementary material for: Interpregnancy intervals and adverse birth outcomes in high-income countries: An international cohort study
Source: PLoS One. 2021 Jul 19;16(7):e0255000. doi: 10.1371/journal.pone.0255000 (PMC8289039; doi:10.1371/journal.pone.0255000)
Supplement: S5 Table — (DOCX) [file pone.0255000.s010.docx]

# **S5 Table**. Sensitivity analysis - Association between interpregnancy interval and adverse birth outcomes for women with ≥3 births/ ≥2 IPI in the between-women analyses* for adjusted for parity, maternal age, time period across the four countries.

| **Outcome by country** | **Interpregnancy interval** | | | | | | |
| --- | --- | --- | --- | --- | --- | --- | --- |
|  | **<6 months** | **6-11 months** | **12-17 months** | **18-23 months** | **24-59 months** | **60-119 months** | ≥**120months** |
| **PTB**  a**OR (95% CI)** | | | | | | | |
| **Australia** | 1.61 (1.55, 1.67) | 1.11 (1.07, 1.15) | 0.97 (0.94, 1.01) | Ref | 1.13 (1.09, 1.16) | 1.53 (1.47, 1.59) | 2.02 (1.87, 2.19) |
| **Finland** | 1.53 (1.44, 1.63) | 1.02 (0.97, 1.08) | 0.95 (0.90, 1.00) | Ref | 1.04 (0.99, 1.09) | 1.31 (1.24, 1.39) | 1.58 (1.43, 1.74) |
| **Norway** | 2.07 (1.94, 2.21) | 1.17 (1.11, 1.24) | 1.01 (0.96, 1.07) | Ref | 1.09 (1.05, 1.14) | 1.45 (1.38, 1.52) | 1.78 (1.65, 1.94) |
| **California** | 1.38 (1.34, 1.42) | 1.15 (1.12, 1.18) | 1.07 (1.04, 1.09) | Ref | 1.09 (1.07, 1.12) | 1.34 (1.30, 1.37) | 1.68 (1.60, 1.78) |
| **Spontaneous PTB** | | | | | | | |
| **Australia** | 1.82 (1.73, 1.92) | 1.21 (1.15, 1.26) | 1.01 (0.96, 1.06) | Ref | 1.09 (1.04, 1.14) | 1.49 (1.41, 1.58) | 1.89 (1.69, 2.11) |
| **Finland** | 1.71 (1.59, 1.84) | 1.14 (1.07, 1.21) | 1.02 (0.95, 1.08) | Ref | 1.09 (1.03, 1.15) | 1.38 (1.29, 1.48) | 1.65 (1.47, 1.85) |
| **Norway** | 2.06 (1.90, 2.23) | 1.20 (1.12, 1.28) | 1.04 (0.97, 1.11) | Ref | 1.02 (0.97, 1.08) | 1.35 (1.26, 1.44) | 1.68 (1.51, 1.88) |
| **California** | 1.65 (1.59, 1.72) | 1.17 (1.13, 1.22) | 1.05 (1.01, 1.09) | Ref | 1.14 (1.10, 1.17) | 1.47 (1.42, 1.53) | 1.89 (1.76, 2.03) |
| **SGA** | | | | | | | |
| **Australia** | 1.10 (1.06, 1.14) | 0.99 (0.96,1.02) | 0.99 (0.96, 1.02) | Ref | 1.18 (1.15, 1.21) | 1.64 (1.59, 1.70) | 2.06 (1.92, 2.21) |
| **Finland** | 1.05(0.96, 1.16) | 0.93 (0.86, 1.00) | 0.92 (0.85, 0.99) | Ref | 1.14 (1.07, 1.22) | 1.52 (1.40, 1.64) | 2.31 (2.05, 2.60) |
| **Norway** | 1.27 (1.19, 1.35) | 1.08 (1.03, 1.13) | 1.00 (0.96, 1.05) | Ref | 1.08 (1.04, 1.12) | 1.37 (1.31, 1.43) | 1.80 (1.68, 1.93) |
| **California** | 1.41 (1.37, 1.45) | 1.07 (1.05, 1.10) | 1.01 (0.98, 1.03) | Ref | 1.10 (1.08, 1.13) | 1.31 (1.27, 1.35) | 1.64 (1.55, 1.74) |

aOR- adjusted odds ratio. CI - confidence interval. PTB - preterm birth. SGA - small for gestational age. *Odds ratios calculated using between-women analyses for women with ≥3 births/ ≥2 IPIs after prognostic score adjustment for maternal age, parity, and year of birth.
