# Supplementary material for: Interpregnancy intervals and adverse birth outcomes in high-income countries: An international cohort study
Source: PLoS One. 2021 Jul 19;16(7):e0255000. doi: 10.1371/journal.pone.0255000 (PMC8289039; doi:10.1371/journal.pone.0255000)
Supplement: S6 Table — (DOCX) [file pone.0255000.s011.docx]

**S6 Table.** Sensitivity analysis –Analysis considering the first three births (parity 0, 1, and 2) in between-women* and within-women** analyses in Australia and Norway.

| **Outcome by country** | **Interpregnancy interval** | | | | | | |
| --- | --- | --- | --- | --- | --- | --- | --- |
|  | **<6 months** | **6-11 months** | **12-17 months** | **18-23 months** | **24-59 months** | **60-119 months** | ≥**120 months** |
| **PTB** | **aOR (95% CI)** | | | | | | |
| **Australia** | | | | | | | |
| Between-women | 1.70 (1.64, 1.77) | 1.11 (1.08, 1.15) | 0.99 (0.96, 1.03) | Ref | 1.18 (1.15, 1.22) | 1.68 (1.62, 1.74) | 2.23 (2.08, 2.39) |
| Within-women | 1.13 (1.04, 1.22) | 1.07 (0.99, 1.13) | 1.02 (0.95, 1.09) | Ref | 1.06 (1.00, 1.13) | 1.30 (1.20, 1.41) | 1.63 (1.39, 1.91) |
| **Norway** | | | | | | | |
| Between-women | 1.99 (1.97, 2.12) | 1.15 (1.10, 1.18) | 1.02 (0.97, 1.06) | Ref | 1.14 (1.10, 1.18) | 1.60 (1.53, 1.67) | 2.02 (1.89, 2.17) |
| Within-women | 0.78 (0.68, 0.88) | 1.00 (0.90, 1.11) | 1.08 (0.98, 1.19) | Ref | 1.08 (1.00, 1.17) | 1.41 (1.29, 1.54) | 1.78 (1.51, 2.08) |
| **Spontaneous PTB** | | | | | | | |
| **Australia** |  |  |  |  |  |  |  |
| Between-women | 1.95 (1.86, 2.05) | 1.21 (1.18, 1.26) | 1.05 (1.00, 1.09) | Ref | 1.17 (1.13, 1.21) | 1.63 (1.55, 1.71) | 2.07 (1.88, 2.28) |
| Within-women | 1.61 (1.45, 1.79) | 1.29 (1.18, 1.42) | 1.12 (1.02, 1.22) | Ref | 0.99 (0.92, 1.08) | 1.11 (1.00, 1.24) | 1.40 (1.12, 1.74) |
| **Norway** | | | | | | | |
| Between-women | 1.98 (1.83, 2.14) | 1.20 (1.13, 1.27) | 1.03 (0.97, 1.09) | Ref | 1.08 (1.03, 1.13) | 1.47 (1.39, 1.55) | 1.85 (1.69, 2.14) |
| Within-women | 1.03 (0.88, 1.21) | 1.11 (0.98, 1.26) | 1.07 (0.95, 1.21) | Ref | 0.93 (0.84, 1.02) | 1.12 (1.00, 1.26) | 1.39 (1.12, 1.72) |
| **SGA** | | | | | | | |
| **Australia** | | | | | | | |
| Between-women | 1.06 (1.02, 1.10) | 0.97 (0.94, 1.00) | 0.98 (0.95, 1.00) | Ref | 1.19 (1.16, 1.21) | 1.71 (1.66, 1.76) | 2.12 (2.00, 2.26) |
| Within-women | 0.88 (0.81, 0.95) | 0.97 (0.92, 1.03) | 1.05 (0.99,1.11) | Ref | 1.03 (1.98, 1.09) | 1.26 (1.17, 1.35) | 1.78 (1.52, 2.08) |
| **Norway** | | | | | | | |
| Between-women | 1.26 (1.19, 1.34) | 1.06 (1.02, 1.11) | 1.02 (0.99, 1.06) | Ref | 1.13 (1.09, 1.16) | 1.44 (1.39, 1.49) | 1.84 (1.76, 1.96) |
| Within-women | 0.82 (0.73, 0.92) | 0.96 (0.88, 1.04) | 0.97 (0.89, 1.05) | Ref | 1.06 (1.00, 1.14) | 1.28 (1.18, 1.38) | 1.59 (1.38, 1.82) |

aOR - adjusted odds ratio. CI - confidence interval. IPI - interpregnancy interval. PTB - preterm birth. SGA - small for gestational age. *Odds ratios calculated using between-women analyses for women with ≥2 births/ ≥1 IPI after prognostic score adjustment for maternal age, parity, and year of birth. **Odds ratios calculated using within-women analyses for women with ≥3 births/ ≥2 IPIs after prognostic score adjustment for maternal age, parity, and year of birth.
