# Supplementary material for: Interpregnancy intervals and adverse birth outcomes in high-income countries: An international cohort study
Source: PLoS One. 2021 Jul 19;16(7):e0255000. doi: 10.1371/journal.pone.0255000 (PMC8289039; doi:10.1371/journal.pone.0255000)
Supplement: S7 Table — (DOCX) [file pone.0255000.s012.docx]

**S7 Table.** Sensitivity analysis – Analysis considering births from 1990 onwards in Norway in between-women* and within-women** analyses (1990-2016).

| **Outcome** | **Interpregnancy interval** | | | | | | |
| --- | --- | --- | --- | --- | --- | --- | --- |
|  | **<6 months** | **6-11 months** | **12-17 months** | **18-23 months** | **24-59 months** | **60-119 months** | ≥**120 months** |
| **PTB** | **aOR (95% CI)** | | | | | | |
| Between-women | 2.06 (1.95, 2.18) | 1.18 91.12,1.23) | 1.01 (0.96, 1.05) | Ref | 1.12 (1.08, 1.16) | 1.52 (1.46,1.58) | 1.92 (1.81, 2.03) |
| Within-women | 0.95 (0.86,1.06) | 1.02 (0.93,1.11) | 1.04 (0.96, 1.14) | Ref | 1.10 (1.02,1.18) | 1.33 (1.22, 1.44) | 1.58 (1.37, 1.82) |
| **Spontaneous PTB** | | | | | | | |
| Between-women | 2.05 (1.91, 2.20) | 1.23 (1.16, 1.30) | 1.02 (0.96, 1.07) | Ref | 1.05 (1.00, 1.10) | 1.40 (1.33, 1.48) | 1.78 (1.64,1.92) |
| Within-women | 1.20 (1.05, 1.38) | 1.11 (0.99, 1.24) | 1.03 (0.92, 1.15) | Ref | 0.91 (0.83, 1.00) | 1.05 (0.95, 1.17) | 1.17 (0.97, 1.40) |
| **SGA** | | | | | | | |
| Between-women | 1.24 (1.18, 1.32) | 1.07 (1.03,1.11) | 1.02 (0.98, 1.06) | Ref | 1.11 (1.08, 1.14) | 1.41 (1.37, 1.47) | 1.82 (1.73, 1.91) |
| Within-women | 0.84 (1.76, 0.93) | 0.96 (0.98, 1.04) | 0.99 (0.92, 1.06) | Ref | 1.06 (1.00, 1.13) | 0.84 (0.76, 0.93) | 1.62 (1.42, 1.85) |

aOR - adjusted odds ratio. CI - confidence interval. IPI - interpregnancy interval. PTB - preterm birth. SGA - small for gestational age. *Odds ratios calculated using between-women analyses for women with ≥2 births/ ≥1 IPI after prognostic score adjustment for maternal age, parity, and year of birth. **Odds ratios calculated using within-women analyses for women with ≥3 births/ ≥2 IPIs after prognostic score adjustment for maternal age, parity, and year of birth.
