# Supplementary material for: Interpregnancy intervals and adverse birth outcomes in high-income countries: An international cohort study
Source: PLoS One. 2021 Jul 19;16(7):e0255000. doi: 10.1371/journal.pone.0255000 (PMC8289039; doi:10.1371/journal.pone.0255000)
Supplement: Attachment — Submitted filename: Response to reviewers_R1_17062021.docx [file pone.0255000.s013.docx]

**Journal formatting requirement**

**Comment #1**. Please ensure that your manuscript meets PLOS ONE's style requirements, including those for file naming. The PLOS ONE style templates can be found at <https://journals.plos.org/plosone/s/file?id=wjVg/PLOSOne_formatting_sample_main_body.pdf> and [https://journals.plos.org/plosone/s/file?id=ba62/PLOSOne_formatting_sample_title_authors_affiliations.pdf](about:blank)

**Comment #2.** PLOS requires an ORCID iD for the corresponding author in Editorial Manager on papers submitted after December 6th, 2016. Please ensure that you have an ORCID iD and that it is validated in Editorial Manager. To do this, go to ‘Update my Information’ (in the upper left-hand corner of the main menu), and click on the Fetch/Validate link next to the ORCID field. This will take you to the ORCID site and allow you to create a new iD or authenticate a pre-existing iD in Editorial Manager. Please see the following video for instructions on linking an ORCID iD to your Editorial Manager account: <https://www.youtube.com/watch?v=_xcclfuvtxQ>

**Comment #3.** We note that you have indicated that data from this study are available upon request. PLOS only allows data to be available upon request if there are legal or ethical restrictions on sharing data publicly. For information on unacceptable data access restrictions, please see <http://journals.plos.org/plosone/s/data-availability#loc-unacceptable-data-access-restrictions>.

In your revised cover letter, please address the following prompts:

3a) If there are ethical or legal restrictions on sharing a de-identified data set, please explain them in detail (e.g., data contain potentially identifying or sensitive patient information) and who has imposed them (e.g., an ethics committee). Please also provide contact information for a data access committee, ethics committee, or other institutional body to which data requests may be sent.

3b) If there are no restrictions, please upload the minimal anonymized data set necessary to replicate your study findings as either Supporting Information files or to a stable, public repository and provide us with the relevant URLs, DOIs, or accession numbers. Please see <http://www.bmj.com/content/340/bmj.c181.long> for guidelines on how to de-identify and prepare clinical data for publication. For a list of acceptable repositories, please see <http://journals.plos.org/plosone/s/data-availability#loc-recommended-repositories>.

We will update your Data Availability statement on your behalf to reflect the information you provide.

**RESPONSE:** We have reviewed the above list, addressing all points. Specifically, we have ensured that our manuscript adheres to the formatting requirement by *PLoS One* (as indicated in the template); confirmed that ORCID ID information was provided for the corresponding author; and provided the data availability statement in the online editorial manager and presented as follows:

**Data availability statement**

*The data underlying this study are not publicly available due to agreements that restrict our ability to share the unit record data on ethical and legal grounds. The data are sensitive, and de-identified but potentially re-identifiable. Requests to access these data can be submitted to the following entities: New South Wales, Australia - NSW Centre for Health Record Linkage website: https://www.cherel.org.au/. Western Australia - Data Linkage Western Australia website: https://www. datalinkage-wa.org.au/. Norway, websites:* [*https://helsedata.no/. Finland*](https://helsedata.no/.%20Finland)*: Social and Health Data Permit Authority Findata website:* [*https://findata.fi/en/*](https://findata.fi/en/)

**Point-by-point responses to reviewers**

**Comments from Reviewer 1**

**Comment #1.** It might be worthwhile to include demographic characteristics stratified by IPI as well as country. Including raw OR results would also help clarify the effect of these demographic variables on this issue.

**RESPONSE:** We agree the reviewer’s suggestion has merit. However, as this was a multi-country study, we thought it is more informative to present demographic characteristics stratified by country in Table 1 so that it is consistent with Table 2. The decision to present results in this way for both cohorts, which were used for the within-women (Table 1) and between-women (S1 Table) analyses, is based on published guidelines:

Hutcheon JA, Moskosky S, Ananth CV, Basso O, Briss PA, Ferré CD, Frederiksen BN, Harper S, Hernández‐Díaz S, Hirai AH, Kirby RS. Good practices for the design, analysis, and interpretation of observational studies on birth spacing and perinatal health outcomes. Paediatric and perinatal epidemiology. 2019 Jan;33(1):O15-24.

**Comment #2**. Socioeconomic status is discussed in the methods, but is not actually presented as an adjusted variable in the results. Further, since records of socioeconomic status vary by country, and do not provide a valid basis for comparison, you could consider dropping discussion and presentation of these variables altogether for a more efficient paper.

**RESPONSE:** We agree with the reviewer and have only included SES adjustment as a supplementary analysis, and have placed results in supplementary material (S3 Table) and limited the description of the related results (page 12) to: *“Additional adjustment for SES resulted in negligible change to the results (S3 Table).”*

**Comments from Reviewer 2**

**Comment #1.** Thank you for inviting me to review this highly interesting and impressive manuscript (MS). As the current Senior scientist of one of the referenced papers (#19) and a citizen of one of the countries involved, it is a privilege to contribute. I also give beforehand credit to the 16 authors for their considerable time and efforts leading up to this MS.

**RESPONSE:** We thank the reviewer for this positive comment on our manuscript, particularly on our presentation of the manuscript. We are happy that our manuscript has been reviewed by a senior scientist in the field who has published on the topic and is knowledgeable of one of the study regions.

**Comment #2.** Authors’ conclusions
In short, the overall and most clinically relevant conclusion is that a standard recommendation of 1½ to 2 years’ interpregnancy interval (IPI) may be open for revision towards a shortening, in particular for women in high income countries. That conclusion is based on a thorough follow up of national birth registry data covering more than 5 million pregnancies in four countries on three continents. By use of a robust design, meticulous and advanced data management and analyses, the authors make a very good case in support of their hypotheses.

**RESPONSE:** Again, we thank the reviewer for the comments.

**Comment #3.** Study outcomes and Gestational age
The study outcomes are a) Overall preterm birth (PTB), b) Spontaneous preterm birth (sPTB), and c) Small-for-Gestational Age (SGA) birth. All of them hinge on an ascertainment of gestational age that is as accurate as possible. The basis of the ‘exposure’ (IPI in months) is birth year of the mothers’ very first offspring and was collected over a period from 1980 to 2017. In most cases, Expected Date of Delivery (EDD) was ascertained by use of ultrasound (ULS). In case such information was unavailable, the peculiar (and hitherto novel) variable ‘expected date of conception’ was ascertained based on the woman’s self-reported menstruation history (LMP). Both EDD methods are probability predictions, but LMP is definitely more imprecise than ULS due to the normal variability of menstrual periods between 21 and 35 days. Moreover, routine ULS was no prenatal routine in Norway until well after a National Consensus conference in 1986.

Hence (1), I suggest a sensitivity analysis that compares births before 1985 and after 1990 to rule out any uncertainties as regards equivalent comparisons of the outcomes under study.

**RESPONSE:** We agree with the reviewer that expected date of conception’s ascertainment through woman’s self-reported menstruation history (LMP) will have some limitations. The number of births that occurred before 1985 (n=17 529, 3.5% of all births for the within-women cohort in Norway) in the sample was small and eventually are less likely to influence the results observed in our study. However, as requested we have now included a sensitivity analysis restricting the cohort for births after 1990 (i.e. 1990-2016) in Norway to compare with our result covering the whole cohort period in Norway (i.e. 1980-2016). As shown below in **Table R1**, the results are relatively unchanged suggesting that error (imprecision) due to using LMP instead of ultrasounds did not affect results. We have now added additional statements in methods (See in page 5, para 1 in statistical analysis) and results (See page 12 para 1 in sensitivity analyses) sections and additional analysis results are provided in S7 Table as a supplementary result in the revised manuscript.

“*...and inclusion of births with gestational ages estimated using LMP by restricting births from 1990 onwards in Norway when ultrasound is being widely used to estimate gestational ages.*”

“*Our analysis restricting births from 1990 onwards in Norway when ultrasound is being widely used to precisely estimate gestational ages did not relatively changed our results (S7 Table).*”

**Table R1.** Adjusted odds ratios (aOR) and 95% confidence intervals (CI) between interpregnancy interval and adverse birth outcomes among births in between-women* and within-women** analyses in Norway comparing results from births in the whole cohort years (1980-2016) and cohorts restricting births after 1990 (1990-2016).

| **Outcome** | | **Interpregnancy interval** | | | | | | |
| --- | --- | --- | --- | --- | --- | --- | --- | --- |
|  |  | **<6 months** | **6-11 months** | **12-17 months** | **18-23 months** | **24-59 months** | **60-119 months** | ≥**120 months** |
| **PTB** | | **aOR (95% CI)** | | | | | | |
| Between-women^$^ | 1980-2016 | 2.06 (1.96, 2.17) | 1.17 (1.12, 1.22) | 1.02 (0.98, 1.06) | Ref | 1.11 (1.08, 1.15) | 1.51 (1.45, 1.57) | 1.91 (1.82, 2.02) |
|  | 1990-2016 | 2.06 (1.95, 2.18) | 1.18 91.12,1.23) | 1.01 (0.96, 1.05) | Ref | 1.12 (1.08, 1.16) | 1.52 (1.46,1.58) | 1.92 (1.81, 2.03) |
| Within-women^$^ | 1980-2016 | 0.97 (0.88, 1.06) | 1.01 (0.93, 1.09) | 1.03 (0.96, 1.12) | Ref | 1.08 (1.02, 1.15) | 1.38 (1.29, 1.49) | 1.71 (1.51, 1.93) |
|  | 1990-2016 | 0.95 (0.86,1.06) | 1.02 (0.93,1.11) | 1.04 (0.96, 1.14) | Ref | 1.10 (1.02,1.18) | 1.33 (1.22, 1.44) | 1.58 (1.37, 1.82) |
| **Spontaneous PTB** | | | | | | | | |
| Between-women^$^ | 1980-2016 | 2.04 (1.92, 2.18) | 1.22 (1.16, 1.28) | 1.04 (0.99, 1.09) | Ref | 1.06 (1.02, 1.10) | 1.40 (1.34, 1.47) | 1.78 (1.64, 1.90) |
|  | 1990-2016 | 2.05 (1.91, 2.20) | 1.23 (1.16, 1.30) | 1.02 (0.96, 1.07) | Ref | 1.05 (1.00, 1.10) | 1.40 (1.33, 1.48) | 1.78 (1.64,1.92) |
| Within-women^$^ | 1980-2016 | 1.21 (1.08, 1.36) | 1.10 (1.00, 1.21) | 1.04 (0.95, 1.14) | Ref | 0.93 (0.86, 1.01) | 1.12 (1.02, 1.23) | 1.29 (1.09, 1.51) |
|  | 1990-2016 | 1.20 (1.05, 1.38) | 1.11 (0.99, 1.24) | 1.03 (0.92, 1.15) | Ref | 0.91 (0.83, 1.00) | 1.05 (0.95, 1.17) | 1.17 (0.97, 1.40) |
| **SGA** |  | | | | | | | |
| Between-women^$^ | 1980-2016 | 1.28 (1.22, 1.35) | 1.08 (1.05, 1.12) | 1.02 (0.99, 1.05) | Ref | 1.11 (1.08, 1.14) | 1.42 (1.37, 1.46) | 1.83 (1.75, 1.92) |
|  | 1990-2016 | 1.24 (1.18, 1.32) | 1.07 (1.03,1.11) | 1.02 (0.98, 1.06) | Ref | 1.11 (1.08, 1.14) | 1.41 (1.37, 1.47) | 1.82 (1.73, 1.91) |
| Within-women^$^ | 1980-2016 | 0.88 (0.81, 0.97) | 0.98 (0.91, 1.05) | 0.97 (0.91, 1.03) | Ref | 1.05 (1.00, 1.11) | 1.26 (1.18, 1.34) | 1.63 (1.45, 1.82) |
|  | 1990-2016 | 0.84 (1.76, 0.93) | 0.96 (0.98, 1.04) | 0.99 (0.92, 1.06) | Ref | 1.06 (1.00, 1.13) | 0.84 (0.76, 0.93) | 1.62 (1.42, 1.85) |

aOR - adjusted odds ratio. CI - confidence interval. IPI - interpregnancy interval. PTB - preterm birth. SGA - small for gestational age. *Odds ratios calculated using between-women analyses for women with ≥2 births/ ≥1 IPI after prognostic score adjustment for maternal age, parity, and year of birth. **Odds ratios calculated using within-women analyses for women with ≥3 births/ ≥2 IPIs after prognostic score adjustment for maternal age, parity, and year of birth.

**Comment #4.** Maternal overall reproductive history Nulliparae are exceptional since they, unlike parous women, have never given birth before. On the other hand, it is a frequent speculation that women tend to repeat the same outcome of consecutive births. In fact, the overall study hypothesis of Ref #19 (above) was to compare outcomes across sibships and therefore, selectively included women who expected their 2nd or 3rd child and whose earlier birth outcomes were known (Bakketeig et al., 1993).
Hence (2), I would like to see some clinical and other background information related to the ‘nullip’ pregnancies, i.e. in addition to the date when the delivery took place. This argument is relevant since any future modification of advice about IPI in a clinical setting normally must take that perspective into account. I have accessed the registration form for all births reported to the Norwegian Birth Registry, the most recent revision of which occurred in 1998. Three important variables that are recorded and should be considered are: a) Pre-eclampsia (varying from slight hypertension to full-blown eclampsia) is a strong predictor of SGA, b) PTB (overall) is recorded as one of the reasons for transfer of the child to the neonatal intensive care unit, and c) the mother’s current occupation (including branch) is recorded and listed as none, part-time or full time. The latter runs counter to the reported “N/A” in Table S1 (maternal occupation)

**RESPONSE:** We acknowledge the reviewer’s comment on the importance of the outcomes/complications of the pregnancies prior to the interval (i.e. pre-eclampsia and PTB) and correlates of socioeconomic status (i.e. occupation) and this is acknowledged in our published protocol [1]. We have classified this issue in our research program as “effect modification”. The primary design for this study was an analysis matched by mothers. We found that conditioning on the outcome/complication of the pregnancy prior to the interval in the matched analysis introduced problems with estimation because for women with two intervals, the outcome/complication of the second pregnancy is also an effect modifier of the effect of IPI for the third pregnancy. Because we could not apply the same primary design and report results comprehensively in the same multi-national study, we selected to publish the results for effect modification by PTB status before the IPI separately [2]. Of interest are the 15 supplementary tables and the mini-commentary that accompanies the article. Our original multi-institutional agreement did not include effect modification by pregnancy complications before the IPI, but we have undertaken a study on the topic using the Australian cohort. This study has been accepted by Paediatric and Perinatal Epidemiology for a special issue that will be released soon.

Briefly, the reviewer implied that recurrence is important, and the results of these studies verify that the reviewer is correct. Recurrence is a very large contributor to risk and a much larger contributor than the interpregnancy interval. Effects do differ by previous preterm birth status (PTB vs term). Relative risks for associations with short IPI were higher for those with births before the interval that were born term, not preterm.

We have now described effect modification by previous PTB in the Discussion section in page 15, para 1 as follows

*“As the primary design for this study was within-women (matched) analysis, conditioning on the outcome (i.e adverse birth outcomes) prior to the interval would introduce problems with estimated effect sizes because for those women with two intervals, the outcome of the second pregnancy would also be an effect modifier of the effect of IPI on the third pregnancy. However, a separate analysis* *using between-women analysis, investigating the effect-modification role of previous PTB* *suggested that previous PTB may* *modify the effect of IPI on PTB in the subsequent pregnancies [53].”*

For SES measurement, although we used proxy variables such as employment or educational status, we did not have near complete information for this variable in Norway. As the reviewer indicated, information for employment status was recorded since 1998 and hence only 36% of our cohort in Norway had information for employment status which led us to limit sensitivity analysis to other sites including Finland where information on employment status was provided. Sensitivity analysis in the other sites showed negligible changes to our main results (See S3 Table).

**Comment #5.** Maternal ethnicity
The overall part of the current study population is most likely Caucasian white, with US Californians as a potential exception. On the other hand, it seems to be a fact that for instance Afro-Americans have a slightly different and left-skewed gestational age profile. This may have an impact on all study outcomes that rely on predicted gestational length.
Hence (3), as a minimum I would like to see a comment about generalizability of study outcomes across ethnicity.

**RESPONSE**: We agree with the reviewer and have now included additional statements in the discussion section to acknowledge this. See page 15, para 1 as follows.

*“**Our study also did not adjust for maternal ethnicity/race due to lack of complete information across cohorts and it is possible that the between-women results are prone to confounding by these variables. Since maternal ethnicity/race would not change between pregnancies, our within-women analysis will account this time-invariant confounder and therefore not adjusting for maternal ethnicity/race would not significantly change within-women results. Nonetheless, generalisability to other study populations might still be affected by within-country heterogeneity in race/ethnicity. However, a Californian study reported that the effect of short IPI on the risks of PTB did not substantially differ between non-Hispanic White and non-Hispanic Black women [2].*

**Comment #6.** Extremely long IPIs and other reproductive aspects
I endorse the discussion related to the influence of IPIs of 60 months (= 5 years) and over. One aspect is the potential change of partner, a result of which is that the fetus is exposed to an entirely novel mix of maternal/paternal epigenetic imprint that in turn may influence the outcome. One example is the ‘return to null’ in terms of an increased risk for preeclampsia (PE; cf. Ref. 43) that add to the effect an advanced maternal age has on PE and other reproductive outcomes.
Hence (4), as PE is risk for SGA, a comment is requested about any potential effect of a consistent fatherhood or not.

**RESPONSE:** Thanks for this important suggestion. We have now added additional discussion on the potential role of partner change and PE. See page 14, para 2 in the discussion section as follows:

“*While partner change is also suggested as a potential factor explaining the association between IPI and adverse pregnancy outcomes, a recent study in Western Australia indicated that partner* *changes between pregnancies did not influence the association between IPI and risk of preeclampsia [45].*”

**Comment #7.** Further, with an observed prevalence of about 1%, the authors claim that there is but a negligible influence on the outcomes under study in pregnancies after assisted reproduction. Their current comment is that the IPI has been inadvertently extended. However, recent data from the Norwegian Birth Registry indicates that close to one in ten pregnancies belongs to that category and should be taken into account in future analyses. On the other hand, this is a situation where the ‘date of conception’ mentioned earlier can be confirmed quite precisely.
Hence (5), please add or modify the current text. You may also consider to pay less attention to the cost issue?

**RESPONSE:** We agree with the reviewer that there appears to be an increasing trend in reproductive technology. It remains unclear as to whether this is sufficiently prevalent to influence the observed results in our study which included births from 1980. A Norwegian study indicated that the prevalence of births using assisted reproduction increased from 0.01% in 1985 to 3.7% in 2014 [3]. The timeline in *Goisis et al*. study covers most of the years in our cohort period and we believe that use of reproductive technology would not affect the results observed in our study. As requested, we have revised our discussion accordingly and also reduced our description regarding the ‘cost of treatment for ART’ leading to longer time between two pregnancies. See the following revised statements in the discussion in page 14, para 3 as follows:

*“Although we expect that only small proportion of women (1-4%) received fertility treatment in the* *cohort periods of this study [49-51] the uptake of reproductive technology is remarkably increasing in some countries [52]* *and likely to follow similar trend in the future, which may suggest that future studies need to account for assisted reproductive technology.”*

**Comment #8** Study design
PLoS ONE is a journal that aims broadly towards a general medical and other scientific readership. My impression and concerns after some 30 years are that the two words ‘retrospective’ and ‘cohort’ used together may (repeat: MAY) seem unfamiliar and contra-intuitive to a number of readers.
Hence (6), I ask the authors to present the study design as in the title and the abstract = ‘...an international longitudinal cohort study. In so doing, it underscores that these are the results of a standard follow up study of a single exposure (= ordinal levels of IPI) and three definite birth outcomes (= PTB, SPTB and SGA). The use of historic data - as shown by how they were collected - are in my view, obvious and sufficient.

**RESPONSE:** We agree with the reviewer and we have updated the description of the design of the study accordingly: *“an international longitudinal cohort study”.*

**Comment #9.** In conclusion, this is an important and most valuable paper that merits publication in PLoS ONE. I enjoyed reading it and have already commended the authors for their initiative and the impressive amount of work they have laid down. Data have been analysed according to state-of-art and the results presented precisely and diligently. I hope my comments may be helpful in the upcoming process, whether they be taken into account or rebutted. My grading advice = Minor revision.

**RESPONSE:** We thank the reviewer for the time taken to assess our manuscript. We have attempted to respond to and accommodate all the reviewer’s suggestions when possible.

**Comments from reviewer #3**

**Comment #1.** In this manuscript the authors combine data from four countries to examine the effects of interpregnancy intervals on adverse outcomes, focusing on preterm birth, spontaneous preterm birth, and small for gestational age. The manuscript is presented in a straightforward manner, and in well-written scientific English. There are a few questions and concerns for the authors to consider.

**RESPONSE:** We thank reviewer for this positive comment on our manuscript, particularly on our presentation of the manuscript.

**Comment #2.** First, with respect to definitions of IPI, the authors give readers a bit more detail. Its a fairly complicated topic actually, on which knowledgeable researchers might disagree. Consider consulting one of two papers from an expert panel convened by the US Office of Population Affairs:

Hutcheon, Jennifer A., Susan Moskosky, Cande V. Ananth, Olga Basso, Peter A. Briss, Cynthia D. Ferré, Brittni N. Frederiksen, Sam Harper, Sonia Hernández-Díaz, Ashley H. Hirai, Russell S. Kirby, Mark A. Klebanoff, Laura Lindberg, Sunni L. Mumford, Heidi D. Nelson, Robert W. Platt, Lauren M. Rossen, Alison M. Stuebe, Marie E. Thoma, Catherine J. Vladutiu, Katherine A. Ahrens, “Good Practices for the Design, Analysis, and Interpretation of Observational Studies on Birth Spacing and Perinatal Health Outcomes”, Paediatric and Perinatal Epidemiology, 33,1 (January 2019), O15-O24. DOI: 10.1111/ppe.12512 PMID: 30311958

Ahrens, Katherine A., Jennifer A. Hutcheon, Cande V. Ananth, Olga Basso, Peter A. Briss, Cynthia D. Ferré, Brittni N. Frederiksen, Sam Harper, Sonia Hernández-Díaz, Ashley H. Hirai, Russell S. Kirby, Mark A. Klebanoff, Laura Lindberg, Sunni L. Mumford, Heidi D. Nelson, Robert W. Platt, Lauren M. Rossen, Alison M. Stuebe, Marie E. Thoma, Catherine J. Vladutiu, Susan Moskosky, “Report of the Office of Population Affairs’ Expert Work Group Meeting on Short Birth Spacing and Adverse Pregnancy Outcomes: Methodological Quality of Existing Studies and Future Directions for Research”, Paediatric and Perinatal Epidemiology, 33,1 (January 2019), O5-O14. DOI:10.1111/ppe.12504 PMID: 30300948

Either of these papers will provide readers with more context than is provided solely by reference 20 on p 5.

**RESPONSE:** Thank you for the suggested references. As noted by the reviewer, there are inconsistencies between studies in how they have defined IPIs. Typically, these differences depend on the use of 1) the date of delivery or conception for either or both of the pregnancies; 2) the mortality status of the pregnancies (e.g. live births, stillbirths, miscarriages); and 3) the minimum week of pregnancy at which births are observed. The approach adopted depends on the data available. The most widely accepted practical definition (also indicated in the second reference suggested by reviewer) is *“the time between delivery of a live birth and the start of the next pregnancy leading to a stillbirth or live birth* [4] *pO7*." Consistent with this definition, we defined IPI as the time between the delivery of one pregnancy that resulted in live- or stillbirth and the conception of the next pregnancy that resulted in live-or stillbirth. Furthermore, for the inclusion of stillbirths in our primary definition, we conducted a sensitivity analysis (*see S4 Table*) restricting to pregnancies resulting in only live births, and found that there were negligible differences in the results which could be attributed to the small (<1%) number of stillbirths in our cohorts. We have now cited the study by *Ahrens et al.* in the revised manuscript.

**Comment #3.** On p 6, first sentence of results, reading Table 1 it appears that the range of women at parity 1 outside the US was 89-93%, not 87-93% as stated. It would be helpful however to clarify how the term parity is used in the study. Are women of parity 1 giving birth to their first-born child, that is, nulliparous, or have they all had a previous live birth. This is unclear from the manuscript.

**RESPONSE:** Sorry for the confusion. We present the statistics based on both the main analysis (Table 1) comprising 89% of women for the within-women analysis and supplementary analysis (S1 Table) comprising 87% of women in the between women analysis were at parity 1 at the time of entry to the cohort. We have now revised the statement to avoid confusion as follows in the result section in page 6, para 1.

*“Approximately 89-93% of women entered the study cohort at parity 1 in Australia, Finland, Norway, and 98% in California for the within-women study (Table 1). For the between-women study, except in California, 87-90% of women entered the study cohort at parity 1 (S1 Table).”*

We have also provided an additional footnote to reflect that parity 1 refers to second births. Given all first births (parity 0) were not applicable for our study (as they are not exposed to an IPI), we have described their characteristics at the time of birth of the second birth after interval in Table 1. For some women who have had any of their births outside the state or country, a participant could have entered the cohort at parity greater than 1 as inclusion requires women with at least two births in the study period irrespective of parity.

**Comment #4**. On a related point however, if we assume that parity=1 are women now giving birth to their second baby, it would be helpful to see the substantive analyses in Table 3 run also only on these births, as that would limit the analysis to birth intervals only for time from first birth to conception of the next pregnancy. As many other studies in the literature do this, more comparable results would then be available.

**RESPONSE**: We agree with the reviewer that IPI and outcome distributions might differ for those births from women in higher-order parity. As requested by the reviewer, we have repeated our analyses presented in Table 3 for the first three births (parity 0, 1 and 2) using both within-women and between-women analysis in Australia and Norway cohorts and as shown below in **Table R2**, our analyses restricting the cohorts to the first three births (parity 0, 1, and 2) did not change our results significantly. We have now added additional statements in the methods (See in page 5, para 1 in statistical analysis) and results (See page 12, para 1 in sensitivity analyses) sections and presented the additional analysis results in S6 Table as a supplementary result in the revised manuscript.

“*Furthermore, we have conducted additional analyses to assess sensitivity of our results for the inclusion of births of from higher-order parity women by restricting our cohort to the first three births (parity 0, 1 and 2) in Australia and Norway...*”

“*Moreover, our additional analysis restricting our cohorts in Australia and Norway to the first three births (parity 0, 1, and 2) for accounting the inclusion of births from women in the higher-order parity did not significantly change the results (S6 Table).*”

**Table R2.** Adjusted odds ratios (aOR) and 95% confidence intervals (CI) between interpregnancy interval and adverse birth outcomes among births from women with Parity 012 in between-women* and within-women** analyses across the four countries.

| **Outcome by country** | | **Interpregnancy interval** | | | | | | |
| --- | --- | --- | --- | --- | --- | --- | --- | --- |
|  |  | **<6 months** | **6-11 months** | **12-17 months** | **18-23 months** | **24-59 months** | **60-119 months** | ≥**120 months** |
| **PTB** | | **aOR (95% CI)** | | | | | | |
| **Australia** | | | | | | | | |
| Between-women | All parity | 1.68 (1.63, 1.73) | 1.13 (1.10, 1.16) | 0.99 (0.97, 1.02) | Ref | 1.16 (1.13, 1.19) | 1.59 (1.55, 1.64) | 2.14 (2.03, 2.27) |
|  | Parity 0, 1, 2 | 1.70 (1.64, 1.77) | 1.11 (1.08, 1.15) | 0.99 (0.96, 1.03) | Ref | 1.18 (1.15, 1.22) | 1.68 (1.62, 1.74) | 2.23 (2.08, 2.39) |
| Within-women | All parity | 1.22 (1.15, 1.29) | 1.08 (1.03, 1.14) | 1.00 (0.95, 1.05) | Ref | 1.06 (1.02, 1.11) | 1.35 (1.27, 1.43) | 1.71 (1.52, 1.92) |
|  | Parity 0, 1, 2 | 1.13 (1.04, 1.22) | 1.07 (0.99, 1.13) | 1.02 (0.95, 1.09) | Ref | 1.06 (1.00, 1.13) | 1.30 (1.20, 1.41) | 1.63 (1.39, 1.91) |
| **Norway** | | | | | | | | |
| Between-women | All parity | 2.06 (1.96, 2.17) | 1.17 (1.12, 1.22) | 1.02 (0.98, 1.06) | Ref | 1.11 (1.08, 1.15) | 1.51 (1.45, 1.57) | 1.91 (1.82, 2.02) |
|  | Parity 0, 1, 2 | 1.99 (1.97, 2.12) | 1.15 (1.10, 1.18) | 1.02 (0.97, 1.06) | Ref | 1.14 (1.10, 1.18) | 1.60 (1.53, 1.67) | 2.02 (1.89, 2.17) |
| Within-women | All parity | 0.97 (0.88, 1.06) | 1.01 (0.93, 1.09) | 1.03 (0.96, 1.12) | Ref | 1.08 (1.02, 1.15) | 1.38 (1.29, 1.49) | 1.71 (1.51, 1.93) |
|  | Parity 0, 1, 2 | 0.78 (0.68, 0.88) | 1.00 (0.90, 1.11) | 1.08 (0.98, 1.19) | Ref | 1.08 (1.00, 1.17) | 1.41 (1.29, 1.54) | 1.78 (1.51, 2.08) |
| **Spontaneous PTB** | | | | | | | | |
| **Australia** | | | | | | | | |
| Between-women | All parity | 1.93 (1.85, 2.01) | 1.22 (1.18, 1.27) | 1.03 (0.99, 1.07) | Ref | 1.13 (1.10, 1.17) | 1.56 (1.50, 1.63) | 1.99 (1.84, 2.16) |
|  | Parity 0, 1, 2 | 1.95 (1.86, 2.05) | 1.21 (1.18, 1.26) | 1.05 (1.00, 1.09) | Ref | 1.17 (1.13, 1.21) | 1.63 (1.55, 1.71) | 2.07 (1.88, 2.28) |
| Within-women^$^ | All parity | 1.66 (1.54, 1.79) | 1.30 (1.22, 1.39) | 1.07 (1.00, 1.14) | Ref | 0.96 (0.90, 1.02) | 1.10 (1.02, 1.19) | 1.28 (1.09, 1.51) |
|  | Parity 0, 1, 2 | 1.61 (1.45, 1.79) | 1.29 (1.18, 1.42) | 1.12 (1.02, 1.22) | Ref | 0.99 (0.92, 1.08) | 1.11 (1.00, 1.24) | 1.40 (1.12, 1.74) |
| **Norway** | | | | | | | | |
| Between-women^$^ | All parity | 2.04 (1.92, 2.18) | 1.22 (1.16, 1.28) | 1.04 (0.99, 1.09) | Ref | 1.06 (1.02, 1.10) | 1.40 (1.34, 1.47) | 1.78 (1.64, 1.90) |
|  | Parity 0, 1, 2 | 1.98 (1.83, 2.14) | 1.20 (1.13, 1.27) | 1.03 (0.97, 1.09) | Ref | 1.08 (1.03, 1.13) | 1.47 (1.39, 1.55) | 1.85 (1.69, 2.14) |
| Within-women^$^ | All parity | 1.21 (1.08, 1.36) | 1.10 (1.00, 1.21) | 1.04 (0.95, 1.14) | Ref | 0.93 (0.86, 1.01) | 1.12 (1.02, 1.23) | 1.29 (1.09, 1.51) |
|  | Parity 0, 1, 2 | 1.03 (0.88, 1.21) | 1.11 (0.98, 1.26) | 1.07 (0.95, 1.21) | Ref | 0.93 (0.84, 1.02) | 1.12 (1.00, 1.26) | 1.39 (1.12, 1.72) |
| **SGA** | | | | | | | | |
| **Australia** | | | | | | | | |
| Between-women^$^ | All parity | 1.10 (1.07, 1.13) | 0.99 (0.97, 1.01) | 0.98 (0.96,1.01) | Ref | 1.18 (1.15, 1.20) | 1.65 (1.61, 1.69) | 2.08 (1.98, 2.19) |
|  | Parity 0, 1, 2 | 1.06 (1.02, 1.10) | 0.97 (0.94, 1.00) | 0.98 (0.95, 1.00) | Ref | 1.19 (1.16, 1.21) | 1.71 (1.66, 1.76) | 2.12 (2.00, 2.26) |
| Within-women^$^ | All parity | 0.94 (0.87, 0.99) | 0.99 (0.95, 1.04) | 1.03 (0.99,1.08) | Ref | 1.04 (1.00, 1.09) | 1.24 (1.17, 1.31) | 1.58 (1.40, 1.78) |
|  | Parity 0, 1, 2 | 0.88 (0.81, 0.95) | 0.97 (0.92, 1.03) | 1.05 (0.99,1.11) | Ref | 1.03 (1.98, 1.09) | 1.26 (1.17, 1.35) | 1.78 (1.52, 2.08) |
| **Norway** | | | | | | | | |
| Between-women^$^ | All parity | 1.28 (1.22, 1.35) | 1.08 (1.05, 1.12) | 1.02 (0.99, 1.05) | Ref | 1.11 (1.08, 1.14) | 1.42 (1.37, 1.46) | 1.83 (1.75, 1.92) |
|  | Parity 0, 1, 2 | 1.26 (1.19, 1.34) | 1.06 (1.02, 1.11) | 1.02 (0.99, 1.06) | Ref | 1.13 (1.09, 1.16) | 1.44 (1.39, 1.49) | 1.84 (1.76, 1.96) |
| Within-women^$^ | All parity | 0.88 (0.81, 0.97) | 0.98 (0.91, 1.05) | 0.97 (0.91, 1.03) | Ref | 1.05 (1.00, 1.11) | 1.26 (1.18, 1.34) | 1.63 (1.45, 1.82) |
|  | Parity 0, 1, 2 | 0.82 (0.73, 0.92) | 0.96 (0.88, 1.04) | 0.97 (0.89, 1.05) | Ref | 1.06 (1.00, 1.14) | 1.28 (1.18, 1.38) | 1.59 (1.38, 1.82) |

aOR - adjusted odds ratio. CI - confidence interval. IPI - interpregnancy interval. PTB - preterm birth. SGA - small for gestational age. *Odds ratios calculated using between-women analyses for women with ≥2 births/ ≥1 IPI after prognostic score adjustment for maternal age, parity, and year of birth. **Odds ratios calculated using within-women analyses for women with ≥3 births/ ≥2 IPIs after prognostic score adjustment for maternal age, parity, and year of birth.

**Comment #5**. Also unclear is whether the analysis file links births into sibships, or if it is a strictly cross-sectional analysis across the years contributed for each country.

**RESPONSE:** Our analysis links births from the same woman using maternal unique identification code. We have now included a statement to clarify that our analysis file links births from the same women. See page 4, para 1 in the Study design and population section

*“These data sources allow to link birth that occurred from the same women using unique identifiers unique to each mother and child.”*

**Comment #6.** In conducting sensitivity analyses, it would be interesting also to control for race/ethnic variation. This probably can't be done in the pooled analysis, but should surely be possible in the US and Australia. Analyses including nativity would also be helpful.

**RESPONSE:** We acknowledge the importance of race/ethnicity. The matched analysis compares births to the same women and therefore ensures sufficient control for race/ethnicity. We acknowledge that confounding by race/ethnicity might affect our unmatched result. However, we were re-assured by a Californian Study indicating that the effect of short IPI on the risks of adverse birth outcomes would not substantially change between non-Hispanic White and non-Hispanic Black women [5]. Please see our response for Reviewer #2, Comment 5.

**Comments from reviewer #4**

**Comment #1.** This manuscript presents some important findings. It uses large samples from 4 developed countries to examine IPI, using both between women and within women analyses. The later is important as it allows the analysis to adjust for unobserved selection bias. That this bias is present is confirmed by the fact that the effects are consistently smaller for the within women analyses. This analysis provides strong support that the current recommendation for a 24 month IPI is not supported for developed countries. I have no concerns with the methods; they are solid and appropriate. The results and conclusions are clearly stated and the conclusions are supported by the results.

**RESPONSE:** We thank the reviewer for this positive comment on our manuscript, particularly on our analysis comparing between women and within women analyses results, and appropriateness of methods, presentation of the results and conclusions in the manuscript.

**Comment #2.** Was very preterm considered as an outcome, in addition to preterm? The consequences of very preterm delivery are many times more serious than moderately preterm delivery. The authors have sufficient sample size to examine this, and it would be an important addition. That said, there is a lot of results in the current manuscript, and adding preterm may be beyond the scope of this study. If so, would encourage the authors to including it in an additional study.

**RESPONSE.** We agree with the reviewer that the consequences of very preterm delivery are more serious than moderately preterm delivery. As this was a multi-country study, some of the analyses must be run at each study site, and consequently the scope for additional analyses not specified in the technical protocol is limited. Nonetheless, we acknowledge the importance of the reviewer’s comment, will consider conducting such a study, and amended the manuscript to include statements in the limitations section as follows on page 15, para 2:

*“**Although associations between IPI and preterm birth might differ by presentation based on timing of delivery (extreme PTB (<28 weeks), very PTB (28-31 weeks), moderate PTB (32-37 weeks)* *[3],* *the scope of this study was limited to separately investigate the association between IPI and PTB classified by clinical presentation (spontaneous and overall PTB).”*

**Comment #3.** The full regression results, including those used to generate the prognostic score, were not presented in the supplementary material. It is appropriate to not report these in the main text, as it would result in huge tables. But, the full results should be made available.

**RESPONSE:** Despite that data analysis have been conducted on different sites as per our technical protocol, we did not have access for beta-coefficients and standard errors for covariates from every site but for the key exposure of outcome for our main analysis results. However, we have now included the parameter estimates for the IPIs of the full regression model (*applicable for meta-analysis*) as an additional file in a CSV.

**Table R3.** Parameter estimates for the association between interpregnancy interval and adverse birth outcomes among births in the between-women* and within-women** analyses across the four countries.

| **Outcome** | **IPI** | **Within-women analysis** | | **Between-women analysis** | |
| --- | --- | --- | --- | --- | --- |
|  |  | **Parameter estimate (beta coefficient)** | **Standard error** | **Parameter estimate (beta coefficient)** | **Standard error** |
| **Australia** | | | | | |
| **PTB** | <6 months | 0.195 | 0.029 | 0.518 | 0.016 |
|  | 6-11 months | 0.080 | 0.026 | 0.122 | 0.014 |
|  | 12-17 months | 0.002 | 0.026 | -0.010 | 0.014 |
|  | 18-23 months |  |  |  |  |
|  | 24-59 months | 0.061 | 0.023 | 0.147 | 0.012 |
|  | 60-119 months | 0.301 | 0.030 | 0.466 | 0.016 |
|  | ≥120 months | 0.535 | 0.061 | 0.762 | 0.029 |
| **sPTB** | <6 months | 0.507 | 0.038 | 0.656 | 0.021 |
|  | 6-11 months | 0.264 | 0.034 | 0.202 | 0.018 |
|  | 12-17 months | 0.066 | 0.034 | 0.030 | 0.018 |
|  | 18-23 months |  |  |  |  |
|  | 24-59 months | -0.041 | 0.031 | 0.124 | 0.016 |
|  | 60-119 months | 0.096 | 0.041 | 0.444 | 0.021 |
|  | ≥120 months | 0.249 | 0.084 | 0.690 | 0.041 |
| **SGA** | <6 months | -0.056 | 0.028 | 0.096 | 0.016 |
|  | 6-11 months | -0.010 | 0.023 | -0.012 | 0.012 |
|  | 12-17 months | 0.033 | 0.023 | -0.018 | 0.012 |
|  | 18-23 months |  |  |  |  |
|  | 24-59 months | 0.043 | 0.021 | 0.164 | 0.010 |
|  | 60-119 months | 0.208 | 0.028 | 0.500 | 0.014 |
|  | ≥120 months | 0.450 | 0.060 | 0.733 | 0.026 |
| **Norway** | | | | | |
| **PTB** | <6 months | -0.031 | 0.047 | 0.722 | 0.026 |
|  | 6-11 months | 0.007 | 0.039 | 0.159 | 0.021 |
|  | 12-17 months | 0.033 | 0.039 | 0.019 | 0.020 |
|  | 18-23 months |  |  |  |  |
|  | 24-59 months | 0.080 | 0.032 | 0.105 | 0.016 |
|  | 60-119 months | 0.323 | 0.037 | 0.411 | 0.019 |
|  | ≥120 months | 0.534 | 0.063 | 0.648 | 0.029 |
| **sPTB** | <6 months | 0.193 | 0.060 | 0.715 | 0.032 |
|  | 6-11 months | 0.095 | 0.049 | 0.196 | 0.026 |
|  | 12-17 months | 0.038 | 0.048 | 0.038 | 0.025 |
|  | 18-23 months |  |  |  |  |
|  | 24-59 months | -0.072 | 0.041 | 0.056 | 0.020 |
|  | 60-119 months | 0.115 | 0.047 | 0.337 | 0.024 |
|  | ≥120 months | 0.251 | 0.083 | 0.570 | 0.038 |
| **SGA** | <6 months | -0.125 | 0.046 | 0.250 | 0.025 |
|  | 6-11 months | -0.022 | 0.034 | 0.077 | 0.018 |
|  | 12-17 months | -0.028 | 0.033 | 0.018 | 0.016 |
|  | 18-23 months |  |  |  |  |
|  | 24-59 months | 0.051 | 0.028 | 0.104 | 0.013 |
|  | 60-119 months | 0.227 | 0.033 | 0.347 | 0.016 |
|  | ≥120 months | 0.486 | 0.057 | 0.606 | 0.025 |
| **Finland** | | | | | |
| **PTB** | <6 months | -0.001 | 0.046 | 0.479 | 0.027 |
|  | 6-11 months | 0.020 | 0.038 | 0.053 | 0.021 |
|  | 12-17 months | -0.050 | 0.038 | -0.013 | 0.021 |
|  | 18-23 months |  |  |  |  |
|  | 24-59 months | 0.001 | 0.034 | 0.112 | 0.019 |
|  | 60-119 months | 0.232 | 0.041 | 0.342 | 0.022 |
|  | ≥120 months | 0.514 | 0.078 | 0.548 | 0.037 |
| **sPTB** | <6 months | 0.275 | 0.054 | 0.569 | 0.031 |
|  | 6-11 months | 0.202 | 0.045 | 0.132 | 0.025 |
|  | 12-17 months | 0.044 | 0.045 | 0.036 | 0.025 |
|  | 18-23 months |  |  |  |  |
|  | 24-59 months | 0.045 | 0.041 | 0.141 | 0.022 |
|  | 60-119 months | 0.299 | 0.050 | 0.369 | 0.026 |
|  | ≥120 months | 0.571 | 0.093 | 0.567 | 0.043 |
| **SGA** | <6 months | -0.410 | 0.067 | 0.091 | 0.040 |
|  | 6-11 months | -0.119 | 0.052 | -0.115 | 0.030 |
|  | 12-17 months | -0.128 | 0.052 | -0.104 | 0.029 |
|  | 18-23 months |  |  |  |  |
|  | 24-59 months | 0.090 | 0.047 | 0.126 | 0.025 |
|  | 60-119 months | 0.313 | 0.055 | 0.435 | 0.029 |
|  | ≥120 months | 0.653 | 0.098 | 0.810 | 0.046 |
| **California** | | | | | |
| **PTB** | <6 months | 0.123 | 0.020 | 0.352 | 0.011 |
|  | 6-11 months | 0.092 | 0.018 | 0.147 | 0.009 |
|  | 12-17 months | 0.070 | 0.018 | 0.062 | 0.009 |
|  | 18-23 months |  |  |  |  |
|  | 24-59 months | 0.003 | 0.016 | 0.111 | 0.008 |
|  | 60-119 months | 0.159 | 0.019 | 0.346 | 0.009 |
|  | ≥120 months | 0.352 | 0.040 | 0.600 | 0.017 |
| **sPTB** | <6 months | 0.291 | 0.032 | 0.512 | 0.015 |
|  | 6-11 months | 0.111 | 0.029 | 0.141 | 0.014 |
|  | 12-17 months | 0.061 | 0.029 | 0.028 | 0.014 |
|  | 18-23 months |  |  |  |  |
|  | 24-59 months | 0.049 | 0.025 | 0.137 | 0.012 |
|  | 60-119 months | 0.300 | 0.030 | 0.416 | 0.013 |
|  | ≥120 months | 0.503 | 0.060 | 0.675 | 0.022 |
| **SGA** | <6 months | 0.229 | 0.022 | 0.353 | 0.011 |
|  | 6-11 months | 0.050 | 0.020 | 0.079 | 0.010 |
|  | 12-17 months | -0.001 | 0.020 | 0.003 | 0.010 |
|  | 18-23 months |  |  |  |  |
|  | 24-59 months | 0.034 | 0.017 | 0.121 | 0.008 |
|  | 60-119 months | 0.157 | 0.022 | 0.314 | 0.010 |
|  | ≥120 months | 0.420 | 0.046 | 0.498 | 0.018 |

IPI - interpregnancy interval. PTB - preterm birth. SGA - small for gestational age. *Parameter (beta coefficient) calculated using between-women analyses for women with ≥2 births/ ≥1 IPI after prognostic score adjustment for maternal age, parity, and year of birth. **Parameter (beta coefficient) calculated using within-women analyses for women with ≥3 births/ ≥2 IPIs after prognostic score adjustment for maternal age, parity, and year of birth

**Selected references**

1.	Marinovich ML, Regan AK, Gissler M, Magnus MC, Haberg SE, Padula AM, et al. Developing evidence-based recommendations for optimal interpregnancy intervals in high-income countries: protocol for an international cohort study. BMJ open. 2019;9(1):e027941. Epub 2019/02/01. doi: 10.1136/bmjopen-2018-027941. PubMed PMID: 30700492; PubMed Central PMCID: PMCPMC6352763.

2.	Marinovich ML, Regan AK, Gissler M, Magnus MC, Håberg SE, Mayo JA, et al. Associations between interpregnancy interval and preterm birth by previous preterm birth status in four high-income countries: a cohort study. BJOG: An International Journal of Obstetrics & Gynaecology. 2020;n/a(n/a). doi: <https://doi.org/10.1111/1471-0528.16606>.

3.	Goisis A, Håberg SE, Hanevik HI, Magnus MC, Kravdal Ø. The demographics of assisted reproductive technology births in a Nordic country. Hum Reprod. 2020;35(6):1441-50. doi: 10.1093/humrep/deaa055. PubMed PMID: 32463875.

4.	Ahrens KA, Hutcheon JA, Ananth CV, Basso O, Briss PA, Ferré CD, et al. Report of the Office of Population Affairs' expert work group meeting on short birth spacing and adverse pregnancy outcomes: Methodological quality of existing studies and future directions for research. Paediatric and perinatal epidemiology. 2019;33(1):O5-O14. Epub 2018/10/09. doi: 10.1111/ppe.12504. PubMed PMID: 30300948.

5.	Lonhart JA, Mayo JA, Padula AM, Wise PH, Stevenson DK, Shaw GM. Short interpregnancy interval as a risk factor for preterm birth in non-Hispanic Black and White women in California. Journal of perinatology : official journal of the California Perinatal Association. 2019;39(9):1175-81. Epub 2019/06/19. doi: 10.1038/s41372-019-0402-1. PubMed PMID: 31209276; PubMed Central PMCID: PMCPMC6713584.
